# Targeting autophagy: polydatin’s role in inducing cell death in AML

**DOI:** 10.3389/fphar.2024.1470217

**Published:** 2024-11-19

**Authors:** Ping Fu, Qing Luo, Chao Wang, Liping Chen, Chang Dong, Ke Yang, Guang Wu

**Affiliations:** ^1^ Department of GCP, The Second Affiliated Hospital, Hengyang Medical School, University of South China, Hengyang, Hunan, China; ^2^ Department of Pharmacy, The Second Affiliated Hospital, Hengyang Medical School, University of South China, Hengyang, Hunan, China; ^3^ COSAY (Guangzhou) Biotech Co., Ltd., Guangzhou, Guangdong, China

**Keywords:** polydatin, AML, Atg5, autophagy, proliferation, apoptosis

## Abstract

Acute myeloid leukemia (AML), a malignant disorder of the hematopoietic system, arises from leukemic stem cells (LSCs) and is the most prevalent form of blood cancer in adults. This study aimed to evaluate the therapeutic potential of polydatin (PD) in AML through *ex vivo* and *in vivo* studies, respectively. This study was prompted by PD’s novel role in enhancing tumor apoptosis and modulating autophagy. *In vitro* studies were conducted using the PD-responsive AML cell line KASUMI-1 and found that PD was able to suppress cell proliferation and induce apoptosis by regulating the autophagy pathway. Subsequently, molecular docking was employed to predict the interaction between PD and Autophagy-related protein 5 (ATG5), a key regulator in the autophagy pathway. It was observed that PD inhibited the ubiquitination of ATG5 and enhanced its protein stability, leading to an increase in ATG5 protein levels and subsequent activation of the autophagy pathway (see in Abstract Graphed). The effectiveness and safety of PD in treating AML were confirmed through *in vivo* experiments using a mouse transplant tumor model, yielding definitive results. Collectively, these results suggest that PD is a promising candidate for the early therapeutic intervention of AML, with a strong potential for clinical application.

## Graphical Abstract

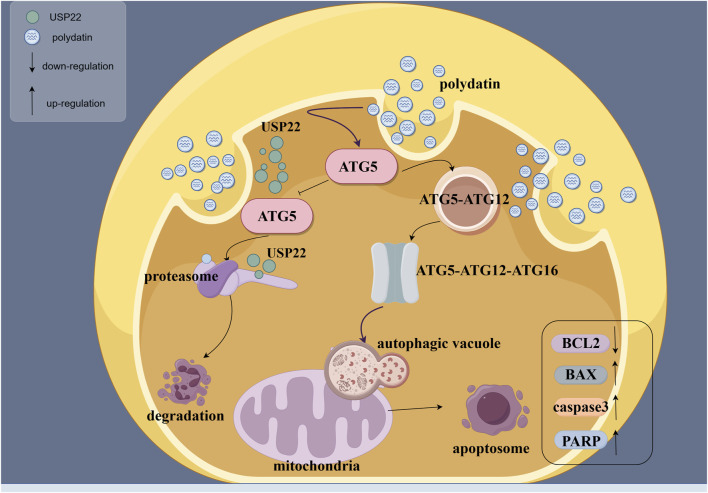

## 1 Introduction

Acute myeloid leukemia (AML) is an aggressive hematological malignancy that is more common in adults (Huan et al., 2022; Wafa et al., 2022). According to the latest world health statistics, there are approximately 474,519 confirmed cases of leukemia worldwide, with 311,497 deaths and a five-year survival rate of 29.5 percent (Siegel et al., 2024). Epidemiological studies have shown that exposure to radiation and chemical substances, myelodysplastic syndrome, myelofibrosis and other hematological or hereditary diseases, as well as immune system defects are the main risk factors for AML (Aitbekov et al., 2022; Guo et al., 2022; Poto et al., 2022). Pharmacological chemotherapy has long been the mainstay of AML treatment (Gomez-Arteaga and Gyurkocza, 2020). However, tumor drug resistance is often an important cause of treatment failure in AML patients, with high tumor recurrence rate and poor prognosis (Mohamed Jiffry et al., 2023; Allison et al., 2022). Therefore, the search for new chemotherapeutic agents is particularly urgent.

Traditional Chinese medicine (TCM) has shown specific advantages in tumor prevention and treatment, including enhancing the body’s vital energy to prevent tumor development, mitigating the adverse effects of tradition interventions such as bone marrow suppression and gastrointestinal reactions, and reducing the recurrence and metastasis of cancer by balancing the body’s systems and ehancing immune surveillance (Ling et al., 2014). Additionally, TCM provides personalized treatment strategies tailored to individual conditions through syndrome differentiation and employs a multi-target approach, leveraging the diverse active components in herbal remedies for a comprehensive disease intervention (Yuan et al., 2022). These findings positioned TCM as a significant complementary therapy in cancer care. PD, extracted from the root and rhizome of *Polygonum cuspidatum Sieb*, which possesses various biological activities including anti-inflammatory, antioxidant, and antiviral effects (Idoudi et al., 2024; Li et al., 2023). Recent research on PD has demonstrated its significant inhibitory effect on tumor therapy, solidifying its potential as a promising anti-tumor medication (Li et al., 2023). Studies have shown that PD inhibits the proliferation of liver cancer cells and induces apoptosis in tumor cells by suppressing Protein kinase B (Akt) phosphorylation (Jiang et al., 2019; Farooq et al., 2023; Enayati et al., 2022); In colon cancer cells, the treatment of PD combined with 5-FU disrupts their mitochondrial function and thus enhances tumor cell chemosensitivity (Bae et al., 2021). Moreover, Guo et al. found in their study of the lung cancer nude mouse transplanted tumor model that PD demonstrated dual effects by alleviating radiation-induced damage to healthy tissue and enhancing radiosensitivity by inhibiting B-cell infiltration in tumor tissue, resulting in a notable increase in apoptosis in lung cancer cells (Guo et al., 2023). It is crucial to recognize that while PD has demonstrated anti-tumor properties in certain cancers, its impact on the progression of acute myeloid leukemia and associated mechanisms remains unknown.

Autophagy, an intracellular process essential for cellular homeostasis and response to external stimuli through the degradation of intracellular substances, has been a subject of investigation in the field of oncology (Wang et al., 2022; Zhao et al., 2022). Relevant literature suggests that activating autophagy may influence tumor progression and treatment outcomes (Ahmadi-Dehlaghi et al., 2023; Zhao et al., 2022; Magnano et al., 2021). ATG5 is essential in the autophagy process as it aids in the creation and breakdown of autophagosomes, thereby impacting the regulation of both classical and nonclassical autophagy pathways (Changotra et al., 2022; Chen et al., 2023). Previous research has demonstrated that the upregulation of ATG5 expression following treatment with chemotherapeutic agents activates the autophagy pathway, leading to the inhibition of tumor cell proliferation and the promotion of apoptosis (Zhu et al., 2019; Bahar et al., 2022; He et al., 2022). This suggests that ATG5 may serve as a promising target for treating tumors.

Drawing on the aforementioned research foundation, the primary objective of this study is to explore the therapeutic efficacy of PD in the treatment of AML and elucidate the precise mechanism involving the ATG5 autophagy pathway through a combination of *in vivo* and *in vitro* experiments. It is anticipated that the results of this research will enhance the theoretical basis for the clinical treatment and alleviation of AML.

## 2 Materials and methods

### 2.1 Cell lines, reagents and drug

Human AML cells: KG-1(No.CCL-246.1), HL-60 (No.CRL-3306), KASUMI-1 (No.CRL-2724), KASUMI-6 (No.CRL-2775), murine AML cell line: C1498 (No.TIB-49), all purchased from American Type Culture Collection (ATCC) Cell Bank, United States. SPF NCG mice, 4-5 weeks old, weighing 16–18 g, were purchased from Beijing Vital River Laboratory Animal Co., Ltd. Animal production license number: SCXK (Beijing) 2021-0011.

Reagents and drugs: fetal bovine serum (No.A5669701), RPMI1640 medium (No.11875119) were purchased from Gibco, United States; penicillin-streptomycin solution (No.C0222), cell counting kit-8 (No.C0038) and BCA protein concentration quantitative kit (No.P0010S) were purchased from Shanghai Beyotime Biotechnology. Hematoxylin-eosin (HE) staining kit (No. G1120) was purchased from Soleibao Biotechnology Company, Beijing, China. 5-bromo-2-deoxyuracil (EDU) kit (No. KGA9602-100), human interleukin-6 (IL-6), IL-1β, Tumor necrosis factor -α (TNF-α) enzyme-linked immunosorbent assay (ELISA) kit (No. KGC1111-48, KGC1103-48, KGC1122-48), flow cytometry Annexin-v-FITC kit (No. KGA1101-100) were purchased from China Jiangsu Keygen Biological Co., Ltd.

Mouse hemoglobin (Hb) ELISA kit (No. LE-M1587) was purchased from Hefei Lyle Biotechnology Co., Ltd., China, and mouse erythropoietin (Epo) ELISA kit (No.XG-E989530) was purchased from Shanghai Xige Biotechnology Co., Ltd., China. human B-cell lymphoma-2 (BCL2) antibody (No.12789-1-AP), human Bcl-2 Associated X protein (BAX) antibody (No. 50599-2-Ig), human Poly ADP-ribose polymerase (PARP) antibody (No.13371-1-AP), ATG5 Monoclonal antibody (No. 66744-1-Ig) and human Cleaved caspase3 antibody (No.19677-1-AP) were purchased from Wuhan Proteintech Biological Co., Ltd. Polydatin (No.HY-N0120A) was purchased from MCE Biological Co., Ltd. The purity of Polydatin (batch number: HY-N0120A-29) was 99.57%.

Instruments: DILITCEN22 desktop centrifuge, Suzhou Beirui Instrument Co., Ltd., China; hBS-ScanX full wavelength microplate reader, Nanjing Detieer Experimental Equipment Co., Ltd., China; dYJ-905 inverted metallographic microscope, Shanghai Dianying Optical Instrument Co., Ltd., China; Invitrogen iBright all-round gel imager, Thermo Fisher Science and Technology Co., Ltd., United States; FACSCanto II flow cytometer, BD Biomedical, United States; 1290 LC-MS, 1290 UHPLC with 6230TOF MS System, Agilent, United States.

### 2.2 Cell culture

The cells were cultured in RPMI1640 medium supplemented with 10% fetal bovine serum and 1% penicillin and streptomycin at 37°C with 5% CO_2_. Upon reaching a cell density exceeding 80%, the cells were harvested by centrifugation and passaged at a ratio of 1:3.

### 2.3 Half maximal inhibitory concentration (IC_50_) assay

KG-1, HL-60, C1498, KASUMI-1, and KASUMI-6 cells were plated in 96-well plates at a density of 8 × 10^3^ cells per well. PD stock solution was prepared by dissolving in dimethyl sulfoxide (DMSO) according to the manufacturer’s instruction. Following a 12-hour incubation period to allow for cell adherence, the PD stock solution was diluted with culture medium to achieve working concentrations of 1, 5, 10, 20, 40, and 100 μM. The cells were then exposed to these varying concentrations of PD for 48 h (Li et al., 2017). Subsequently, 10 μL of cell counting kit 8 (CCK-8) solution was added to each well, followed by a 2-hour incubation in darkness. Absorbance at a wavelength of 450 nm was measured using an enzyme labeling instrument, and the IC50 value was calculated using GraphPad Prism software.

### 2.4 CCK-8 assay

The KG-1, HL-60, C1498, KASUMI-1, and KASUMI-6 cells were seeded in 96-well plates at a density of 3 × 10³ per well. After 12 h of adherence, the cells were treated with PD (0, 10, 20, and 40 μM), respectively, with 5 replicate wells in each group. At 24, 48, 72, and 96 h after drug treatment, 10 μL of CCK-8 solution was added to each well, which was then incubated in the dark for 2 h. The absorbance was then measured at a wavelength of 450 nm. The cell proliferation rate was calculated according to the following formula: The cell proliferation rate is calculated as follows: (Absorbance value of experimental group - Absorbance value of blank well)/(Absorbance value of control group − Absorbance value of blank well) × 100%.

### 2.5 RT-qPCR assay

KG-1, HL-60, C1498, KASUMI-1 and KASUMI-6 were seeded in 6-well plates at a density of 1 × 10^6^ per well. After adherent for 12 h, the cells were treated with PD (0, 10, 20, and 40 μM), respectively. After 48 h, the total RNA was extracted with Trizol. cDNA was synthesized using a reverse transcription kit according to the manufacturer’s instructions. RT-qPCR was then conducted with a SYBR Prime Script RTPCR kit utilizing custom primers (in Supplementary Table S1). The relative expression levels of mRNA were quantified using the 2^∧^^−ΔΔCt^ method.

### 2.6 Cell treatment

KASUMI-1 cells were seeded in 96-well plates (or 6-well plates) at a density of 3 × 10^3^ (or 1 × 10^6^) per well. After 12 h of attachment, the cells were treated with PD (0, 10, 20, and 40 μM) for 48 h, respectively. After treatment, the relevant experimental tests were performed. The CCK-8 experimental steps were performed according to the above description.

### 2.7 EDU staining

The EDU assay was carried out according to the protocol from the EDU kit manual after treatment. In summary, the EDU reagent was diluted with culture medium to create a working solution, and then was added to the cells for incubation for 6 h. After the incubation, 4% paraformaldehyde solution was added to fix the cells, and 1% Triton X-100 solution was used for membrane penetration. The Click-iT EdU reaction reagent was prepared according to the EDU kit manual and added to the cell culture plate for further incubation for 30 min. Finally, the cells were observed under a fluorescence inverted microscope, and photographs were taken for record.

### 2.8 Flow cytometry assay

Following drug treatment, the procedures outlined in the Annexin V/PI Apoptosis Detection Kit manual were adhered to. This included the collection and washing of cells and supernatant with PBS solution three times, followed by the addition of 5 μL of Annexin V solution and 5 μL of PI solution. Subsequently, the samples were thoroughly mixed and incubated at room temperature in the absence of light for 15–30 min. The samples were then analyzed using appropriate equipment, and the resulting data were processed utilizing Flow jo10.0 software.

### 2.9 Western blot assay

The total protein of the cells was extracted and quantified using a BCA kit after treatment. Subsequently, according to the sample order, 40 μg samples were fractionated using 10% SDS-polyacrylamide gel electrophoresis (PAGE) and subsequently transferred onto a PVDF membrane. Following blocking with 5% non-fat milk, the membrane was incubated overnight at 4°C with the respective primary antibody solution, which was diluted at a ratio of 1:1000. This was followed by a 5-hour incubation with the appropriate secondary HRP-conjugated antibodies (1:8000). Ultimately, the protein signal was visualized using an ECL detection kit, and the strip’s gray value was analyzed using ImageJ software.

### 2.10 ELISA assay

After the cells were treated with drugs, the operation was performed according to the instructions of the ELISA detection kit. In short, the cell supernatant solution was collected and added to the pre-coated well plate. After the incubation, the antibody labeled with the enzyme was washed and added to continue the incubation with the specific antibody. The color reaction was performed, and the absorbance was measured by a microplate reader.

### 2.11 Mechanism analysis

In terms of mechanism analysis, the cells were divided into 0 μM group, 20 μM PD group, ATG5 autophagy inhibitor (agent-82,10 μM) group, and 20 μM PD + ATG5 autophagy inhibitor combination group (agent-82,10 μM) group. After 48 h of treatment, the cells were tested according to the CCK-8, EDU, ELISA, Western blot and flow cytometry operation steps described above.

### 2.12 Animal assay

NCG mice used to construct KASUMI-1 subcutaneous transplantation tumor model. All mice were housed in a sterile mouse house environment, alternating day and night every 12 h, and were given adequate food and drinking water. After 1 week of adaptation, the AML model was established by subcutaneous injection of KASUMI-1 cells (2 × 10^7^) in the right limb of mice. When the tumor volume reached 100 mm^3^, the mice were treated with drugs. PD stock solution was dissolved in 0.9% saline solution to prepare the treatment solutions. The mice in each experimental group received the PD solution via intragastric gavage once daily at dosages of 50 mg/kg, 100 mg/kg, and 200 mg/kg, respectively. The control group was given an equal amount of DMSO diluted in saline solution (Zhang et al., 2019; Li et al., 2023). During the treatment, the tumor volume and the body weight of the mice were measured every 3 days. The tumor volume was calculated according to the formula V = L × W2/2, where L represents the tumor length and W represents the tumor width. After 4 weeks, peripheral blood was extracted from the tail vein of mice, and 2 mL was mixed with 38 mL Turk blood diluent, and then the white blood cell count (WBC) was observed under a microscope. The remaining blood was centrifuged at 12,000 rpm for 5 min, and the supernatant was collected. The expression levels of hemoglobin (Hb) and erythropoietin (Epo) were detected using a whole blood automatic analyzer. After that, the mice were euthanized, and the lung, kidney, spleen and liver tissues of the mice were collected, and then the tissue samples were stained with hematoxylin and eosin. All experimental animals were euthanized for cervical dislocation with a high concentration of CO_2_, and the animal experiments were approved by the Ethics Committee for the Institutional Animal Care and Ethics Committee of The Second Affiliated Hospital, University of South China (Animal Ethics 230319).

### 2.13 HE staining

After the lung, kidney, spleen and liver tissues of mice were fixed and embedded, they were cut into 6 μm slices by frozen section machine. The operation was carried out according to the instructions of HE staining kit, and the pathological structure of the tissues was analyzed by taking photos under the microscope.

### 2.14 Molecular docking

The three-dimensional structure of PD and ATG5 protein was obtained by PubChem. The active binding sites of PD and ATG5 were defined by AutoDock software, and the network was generated.

### 2.15 Pharmacokinetic analysis

The real-time drug concentration of PD in the blood of healthy mice was analyzed by high-performance liquid chromatography (HPLC), with six mice in each group undergoing alternate blood collection. The blood was collected at the following time points: 0 min, 5 min, 15 min, 30 min, 1 h, 2 h, 4 h, 8 h, 16 h, and 24 h. The following parameters were used for the determination: flow rate: The flow rate was set at 0.1 mL/min, the temperature at 25°C, the column at Bonus-PR, and the mobile phase comprised 20 mM sodium phosphate buffer (pH2.5)-acetonitrile (90:10).

### 2.16 Statistical analysis

All experiments were repeated more than 3 times, and data analysis was performed using GraphPad Prism (version 9.0). All results are presented as mean ± standard error (SEM). The difference between the two groups was performed using a 2-tailed Student's t-test. One-way analysis of variance (ANOVA) was used for comparison between multiple groups. *P* < 0.05 was considered significant.

## 3 Results

### 3.1 Effects of PD on drug sensitivity and ATG5 expression in AML cell lines

**FIGURE 1:**
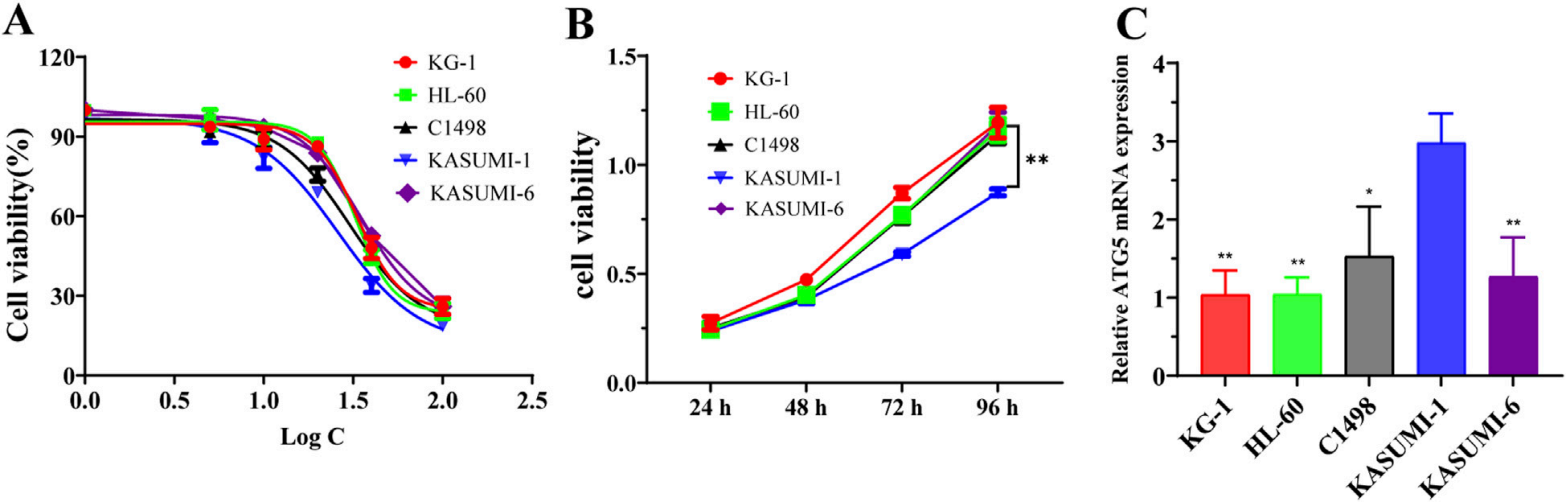
Effects of polydatin on drug sensitivity and ATG5 expression in AML cell lines **(A)**. The IC50 value of polydatin on KG-1, HL-60, C1498, KASUMI-1 and KASUMI-6 cells were determined using the CCK-8 assay. **(B)** The cell viability of polydatin on KG-1, HL-60, C1498, KASUMI-1 and KASUMI-6 cells were assessed using the CCK-8 assay. Compared with KASUMI-1, ^**^ *P* < 0.01. **(C)** The ATG5 mRNA expression of KG-1, HL-60, C1498, KASUMI-1 and KASUMI-6 cells were detected using the RT-qPCR assay. Compare with KASUMI-1 cell, ^*^ *P* < 0.05, ^**^ *P* < 0.01.

The study investigated the drug sensitivity and cytotoxic effects of PD on acute myeloid leukemia (AML) cell lines. The IC50 values of PD for KG-1, HL-60, C1498, KASUMI-1 and KASUMI-6 cells were determined to be 33.21, 32.39, 30.09, 25.88, and 35.02, respectively (Figure 1A). PD exhibited a reduction in cell viability in AML cells, with the KASUMI-1 cell line demonstrating the lowest proliferative viability and highest drug sensitivity (Figure 1B). Furthermore, RT-qPCR analysis revealed a significant upregulation of ATG5 expression in KASUMI-1 cells (Figure 1C). This resulted in the choice of the KASUMI-1 cell line for subsequent experimental investigations.

### 3.2 PD inhibits the proliferation and the inflammatory factors expression of AML cells *in vitro*

**FIGURE 2:**
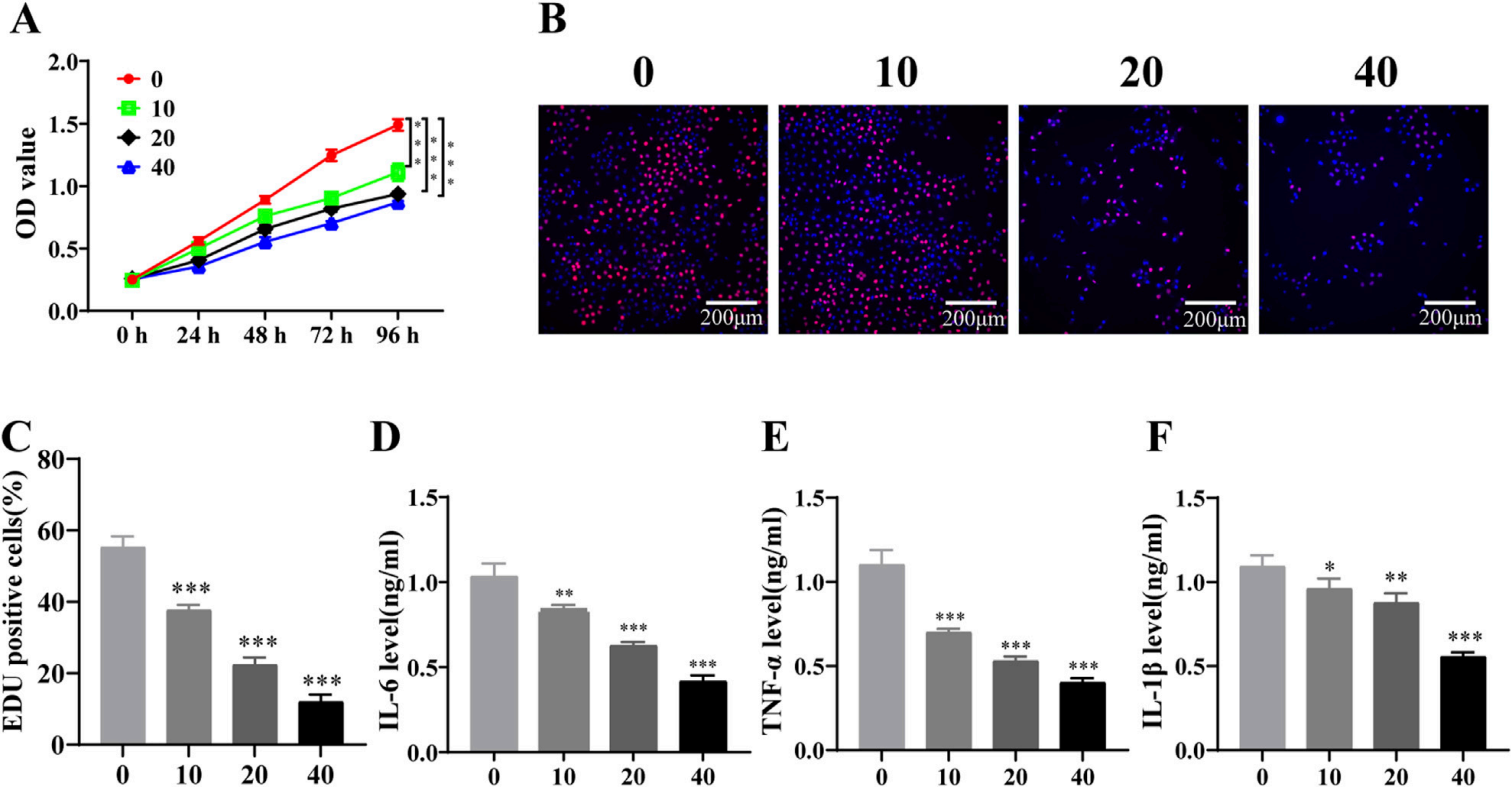
Polydatin inhibits the proliferation of AML cells and the expression of inflammatory factors *in vitro* **(A)**. CCK-8 assay was used to detect the effect of different concentrations of polydatin on the proliferation of KASUMI-1 cells; **(B)**. EDU assay was used to detect the effect of different concentrations of polydatin on the proliferation of KASUMI-1 cell; **(C)**. Statistical analysis of EDU results; **(D–F)** ELISA was used to detect the expression levels of IL-6, TNF-α and IL-1β in different concentrations of polydatin. ^*^ *P* < 0.05; ^**^ *P* < 0.01; ^*^ *P* < 0.001.

CCK-8 assay demonstrated that PD reduced KASUMI-1 cell viability in a dose-dependent manner at different times (Figure 2A). And the analysis of EDU assay showed that the red fluorescence intensity of the cells was significantly lower than that of the control group with the increase of PD concentration (Figures 2B, C), indicating that PD significantly inhibited KASUMI-1 cell proliferation. Additionally, the levels of IL-6, TNF-α, and IL-1β expression were assessed following treatment with PD. The results demonstrated a clear dose-dependent reduction in the expression of IL-6, TNF-α, and IL-1β (Figures 2D–F). These findings suggest that PD has the potential to effectively suppress the proliferation and inflammatory factor expression of AML cells in an *in vitro* setting.

### 3.3 PD induces apoptosis of AML cells *in vitro*

**FIGURE 3:**
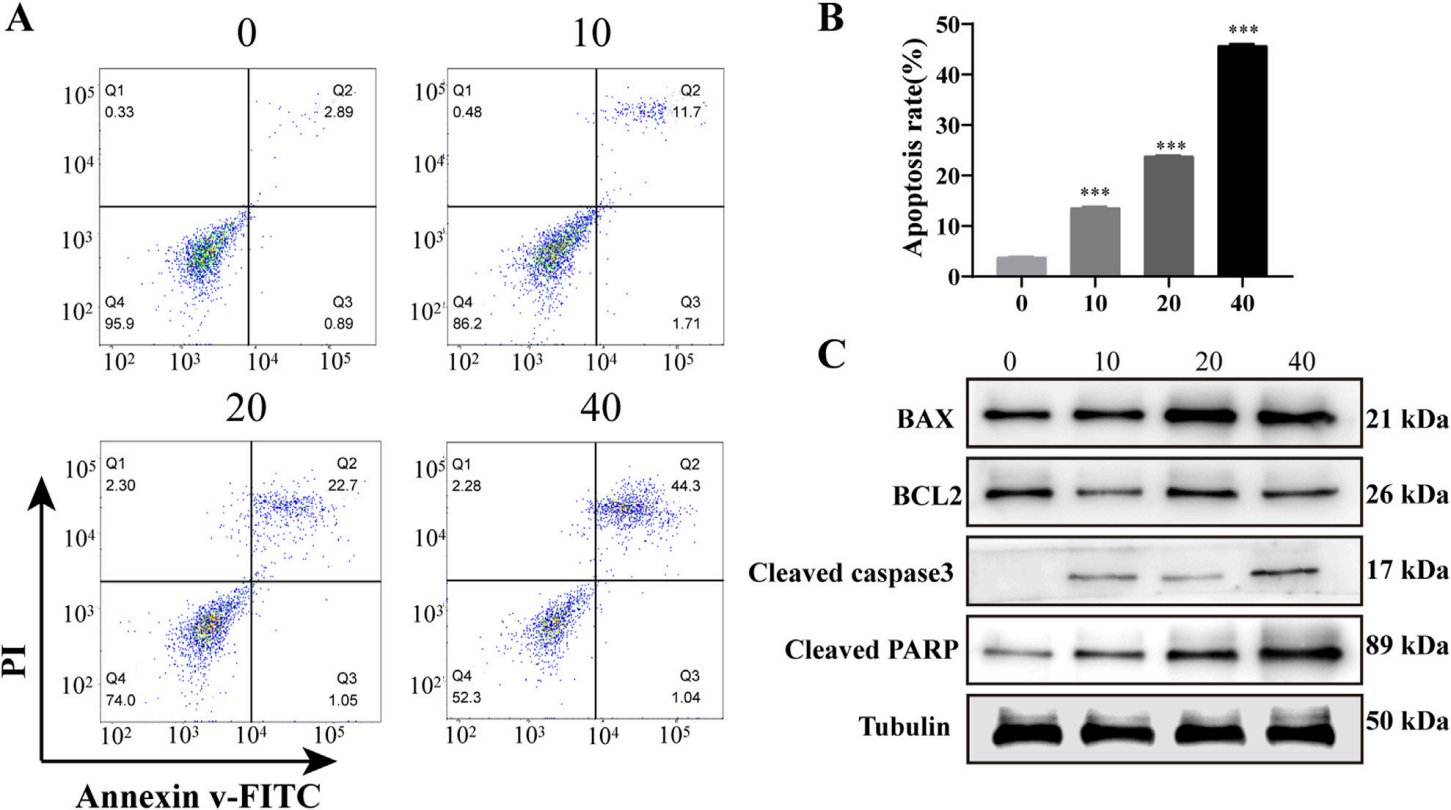
Polydatin induces apoptosis of AML cells *in vitro* **(A)**. Flow cytometry was used to detect the effect of different concentrations of polydatin on the apoptosis of KASUMI-1 cells; **(B)**. Statistical analysis of flow cytometry apoptosis test results; **(C)**. Western blot was used to detect the effects of different concentrations of polydatin on apoptosis-related proteins in KASUMI-1 cells; ^***^ *P* < 0.001.

To assess the impact of PD on the apoptosis of AML cells in an *in vitro* setting, flow cytometry and western blot experiments were conducted subsequent to PD administration. The findings indicated a notable increase in the rate of apoptosis in KASUMI-1 cells following PD treatment, as determined through flow cytometry analysis, in a manner that was dependent on the dosage administered (Figures 3A, B). Consistently, western blot analysis demonstrated that PD incubation decreased the expression of the anti-apoptotic protein Bcl-2, while increasing the expression levels of the pro-apoptotic signals Bax, cleaved caspase-3, and cleaved PARP (Figure 3C).

### 3.4 PD activates ATG5-mediated autophagy pathway *in vitro*

**FIGURE 4:**
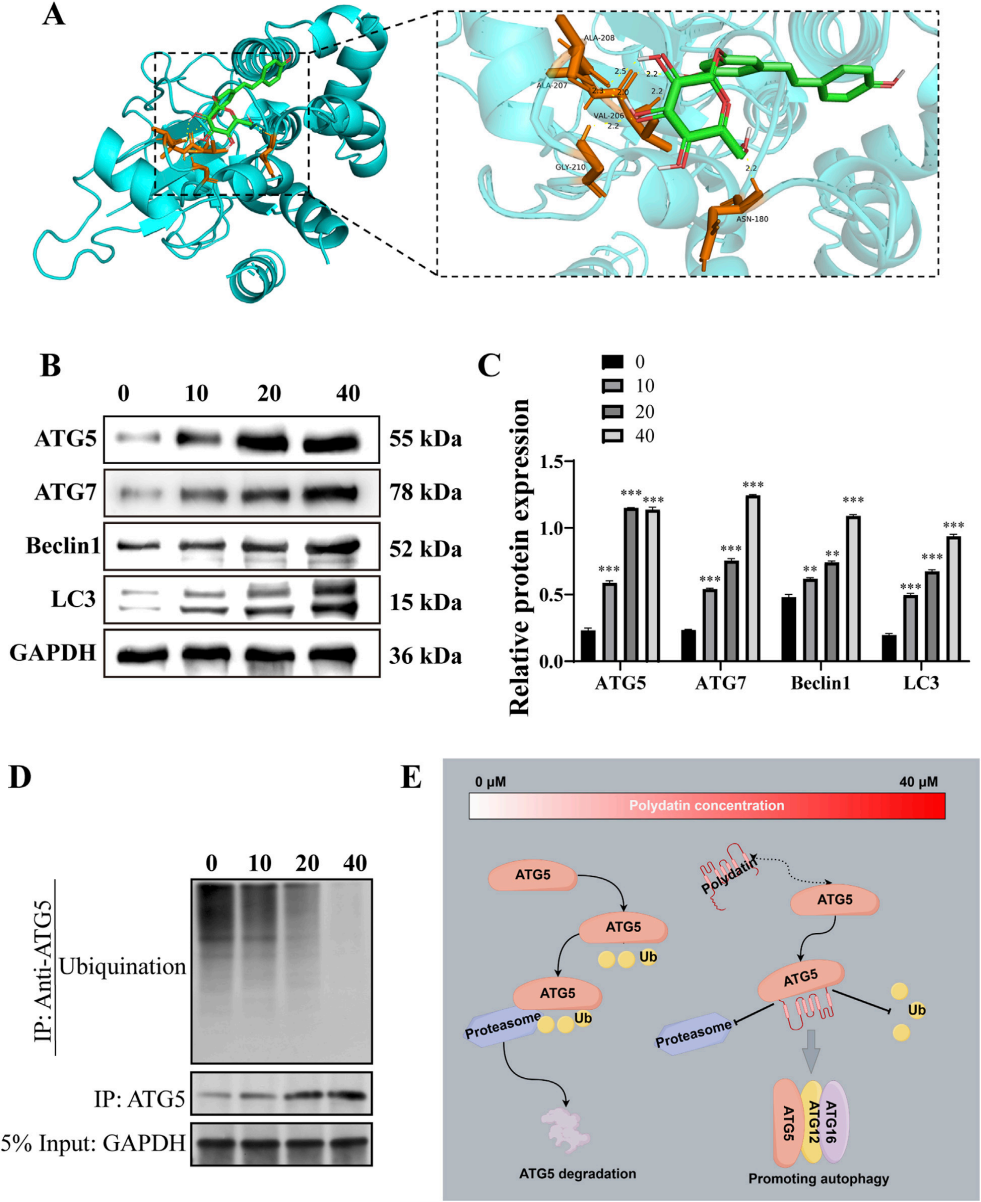
Polydatin activates ATG5-mediated autophagy pathway *in vitro* **(A)**. Molecular docking diagram of polydatin and ATG5; **(B)**. Western blot was used to detected the effect of polydatin on the expression of autophagy-related proteins; **(C)**. Statistical analysis of Western blot results. **(D)** Western-Blot analysis the level of ATG5 ubiquitination in KASUMI-1 cells treated with 0, 10, 20, 40 μM Polydatin; **(E)** Schematic illustration of the molecular mechanism of polydatin inhibits ATG5 ubiquitination to promote autophagy ^*^ *P* < 0.05; ^**^ *P* < 0.01; ^*^ *P* < 0.001.

The Autodock software was utilized to predict the binding activity between PD and ATG5. The analysis revealed multiple amino acid binding sites and binding energies below −5 kJ/mol (Figure 4A), suggesting a strong binding affinity between PD and ATG5. Furthermore, the results indicated that ATG5 may serve as a potential target for PD. Subsequent western blot analysis demonstrated a dose-dependent induction of autophagy in KASUMI-1 cells following PD treatment, as evidenced by the upregulation of ATG5, ATG7, Beclin1, and Microtubule-associated protein 1A/1B-light chain 3 (LC3) (Figures 4B, C). Various concentrations of PD have been found to have a notable impact on the ubiquitination process of ATG5, with a concentration of 40 μM demonstrating a significant reduction in the ubiquitination level of ATG5 (Figure 4D). The collective findings suggest that PD plays a role in decreasing the ubiquitination level of ATG5, consequently enhancing the protein stability of ATG5, increasing the expression of ATG5, and facilitating the initiation of autophagy (Figure 4E).

### 3.5 PD exerts anti-AML tumor effect by regulating ATG5-mediated autophagy pathway

**FIGURE 5:**
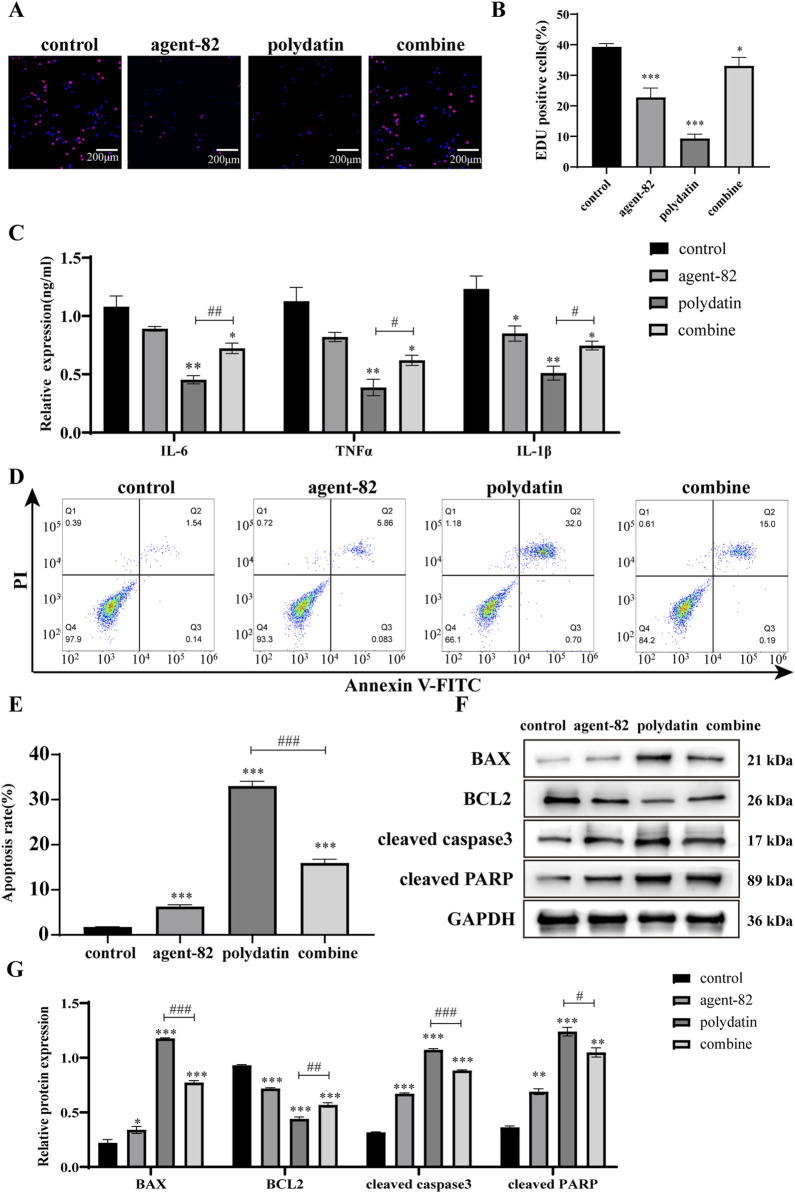
Polydatin exerts anti-AML tumor effect by regulating ATG5-mediated autophagy pathway **(A)**. EDU was used to detect the effect of polydatin combined with agent-82 on the proliferation of KASUMI-1 cells. **(B)** Statistical analysis of EDU results; **(C)** ELISA was used to detect the effect of polydatin combined with agent-82 on the expression of IL-6, IL-1β and TNF-α in KASUMI-1 cells. **(D)** Flow cytometry was used to detect the effect of polydatin combined with agent-82 on the apoptosis of KASUMI-1 cells; **(E)** Statistical analysis of flow cytometry apoptosis test results; **(F)** Western blot was used to detect the effect of polydatin combined with agent-82 on apoptotic proteins in KASUMI-1 cells; **(G)** Statistical analysis of apoptotic protein expression results. Compared with control group, ^*^ *P* < 0.05; ^**^ *P* < 0.01; ^*^ *P* < 0.001; compared with polydatin group, ^#^ *P* < 0.05; ^##^ *P* < 0.01; ^###^ *P* < 0.001.

To elucidate the precise mechanism by which PD acts against AML tumors *in vitro*, functional recovery experiments were conducted utilizing ATG5 inhibitors. The suppressive impact of PD on EDU positivity in KASUMI-1 cells was attenuated when co-administered with an ATG5 inhibitor (agent-82) (Figures 5A, B). ELISA analysis revealed that the combined treatment of agent-82 and PD reversed the inhibitory effects of PD on the expression of IL-6, IL-1β, and TNF-α (Figure 5C). Flow cytometry results indicated that the co-treatment of agent-82 and PD led to a significant decrease in the apoptosis rate of KASUMI-1 cells compared to those treated with PD alone (Figures 5D, E). Additionally, the combined treatment reversed the upregulation of pro-apoptotic proteins Bax, cleaved caspase3, and PARP, as well as the downregulation of the anti-apoptotic protein Bcl2 induced by PD treatment alone (Figures 5F, G).

**FIGURE 6:**
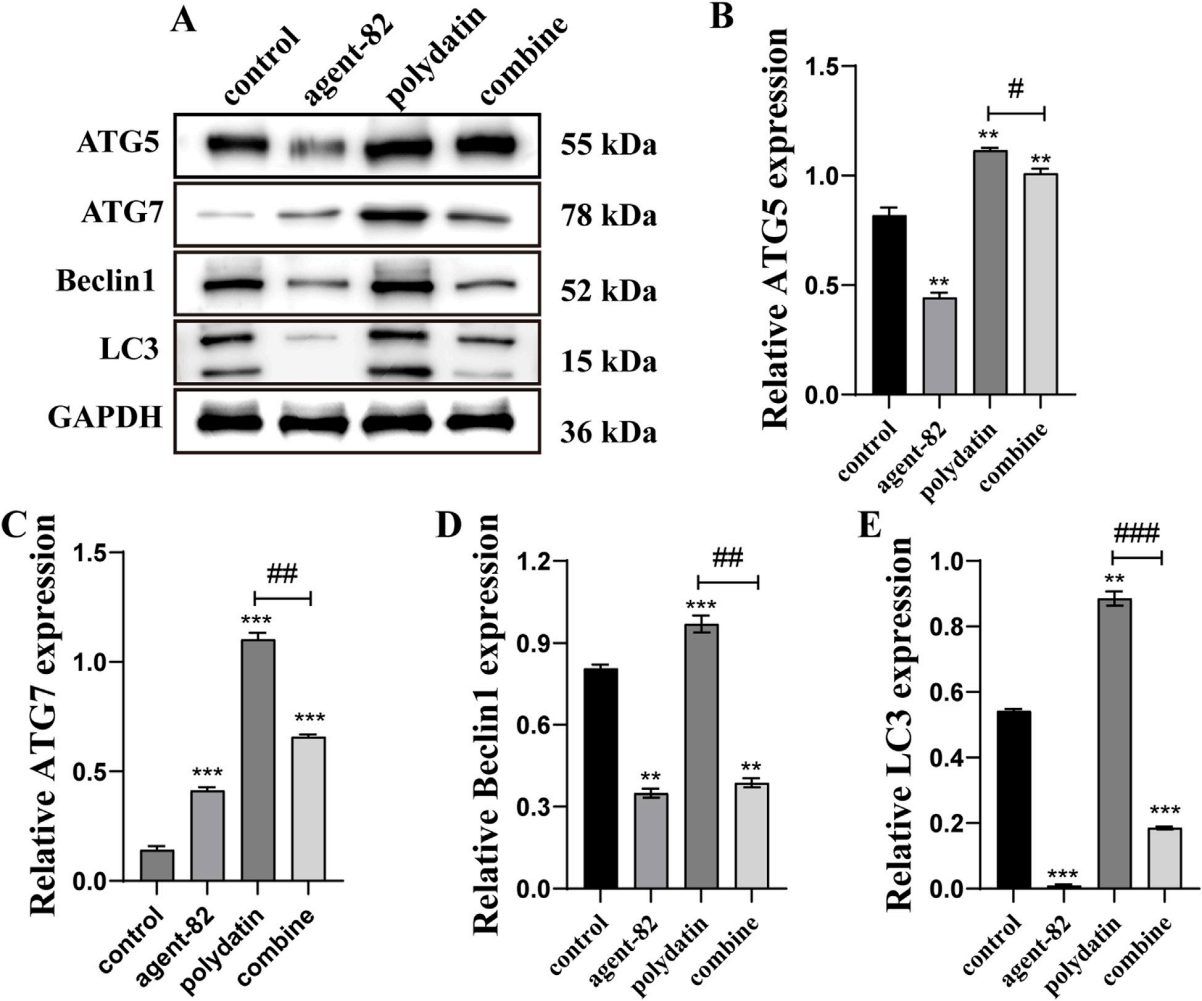
ATG5 inhibitor reversed polydatin-induced autophagy in AML cells. **(A)** Western blot was used to detect the effect of different concentrations of polydatin on autophagy protein in KASUMI-1 cells; **(B**–**E)** Statistical analysis of the results of western blot detection of the effect of polydatin on the expression of autophagy protein. Compared with control group, ^*^ *P* < 0.05; ^**^ *P* < 0.01; ^*^ *P* < 0.001; compared with polydatin group, ^#^ *P* < 0.05; ^##^ *P* < 0.01; ^###^ *P* < 0.001.

Furthermore, we conducted additional experiments to determine if agent-82 could reverse the autophagy-inducing effects of PD on KASUMI-1 cells. Western blot analysis revealed a significant decrease in the expression of ATG5 following treatment with agent-82, as well as a decrease in the expression of other autophagy-related proteins including ATG7, Beclin1, and LC3 when compared to the control group (Figures 6A, B). Interestingly, when PD was combined with agent-82 treatment, the expression of ATG5, ATG7, Beclin1, and LC3 was further suppressed (Figures 6B–E). The aforementioned findings indicate that PD may potentially act as an anti-AML agent through the modulation of the ATG5-mediated autophagy pathway.

### 3.6 PD inhibits AML tumor growth *in vivo*

**FIGURE 7:**
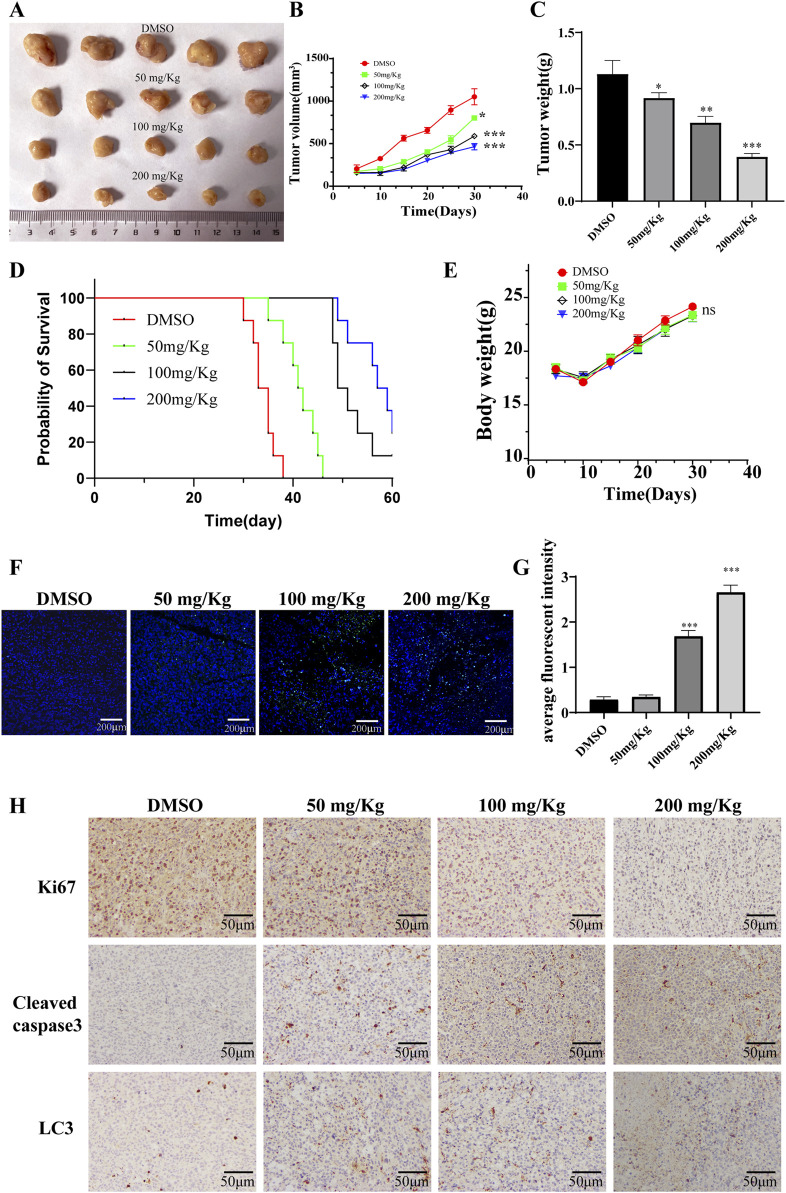
Polydatin inhibits the growth of AML tumor cells *in vivo*. **(A)** Tumor gross appearance; **(B)** Average tumor volume; **(C)** Average tumor weight. **(D)** The survival curve of leukemia-bearing mice calculated by Kaplan-Meier estimate. **(E)** Body weight changes of mice; **(F)** The level of apoptosis was detected by immunofluorescence; **(G)** Statistical analysis of immunofluorescence results. Compare with DMSO group, ^*^ *P* < 0.05; ^**^ *P* < 0.01; ^*^ *P* < 0.001; **(H)** Immunohistochemical staining for Ki67, Cleaved caspase 3, LC3 in tumor tissues at different doses.

The impact of PD on acute myeloid leukemia (AML) was further explored through *in vivo* studies utilizing NCG mice xenografts harboring KASUMI-1 cells. Analysis of Figures 7A–C indicated a significant reduction in tumor growth, volume, and weight following PD treatment. Kaplan-Meier survival analysis demonstrated a higher survival rate in mice treated with PD compared to the control group receiving DMSO throughout the 30-day observation period (Figure 7D). Interestingly, no significant difference in body weight changes was observed between the DMSO and PD treatment groups (Figure 7E). Additionally, the immunofluorescence findings demonstrated a significant induction of apoptosis in AML cells *in vivo* by PD (Figures 7F, G). Immunohistochemical analyses further validated the upregulation of Cleaved caspase3 and LC3 proteins, and downregulation of Ki67 protein in AML cells *in vivo* following PD treatment (Figure 7H). These results suggest that PD exhibits anti-tumor properties through the stimulation of cell autophagy in AML nude mouse models, without causing evident toxicity or adverse effects in mice.

### 3.7 PD improves AML tumor-related blood indicators *in vivo*

**FIGURE 8:**
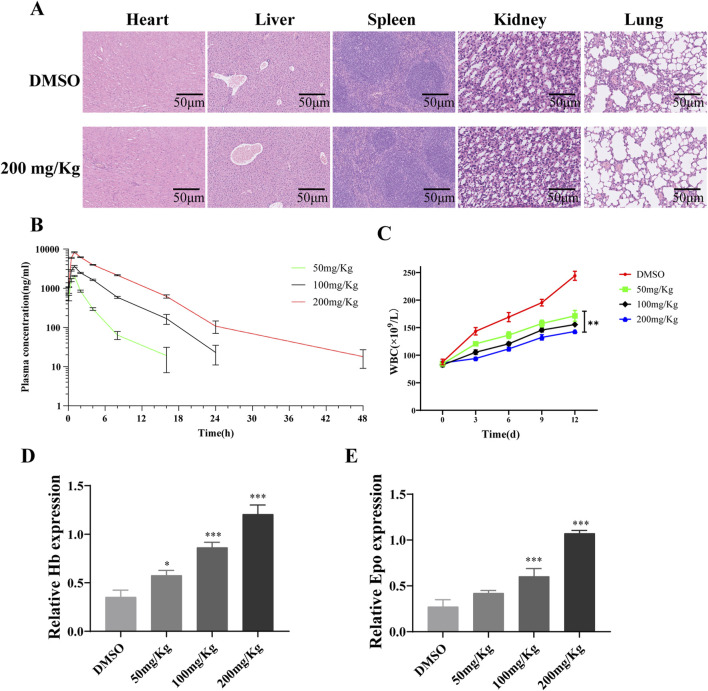
Polydatin improves AML tumor-related blood indicators *in vivo* **(A)** H&E staining for liver, spleen, kidney, lung and heart tissue sections from the DMSO or polydatin treated mice; **(B)** Analysis diagram of drug metabolism of polydatin *in vivo*; **(C)** WBC counting of leukemia-bearing mice; **(D, E)** ELISA was used to detect the effect of polydatin on the expression of Hb and Epo *in vivo*. ^*^ *P* < 0.05; ^**^ *P* < 0.01.

Hematoxylin and eosin staining indicated that PD did not have a significant impact on the histological changes of the heart, liver, spleen, kidney, and lung compared to the DMSO group (Figure 8A). A pharmacokinetic analysis was conducted to assess the metabolism of different doses of PD in mice (Figure 8B). Consistent with its inhibitory effects on AML cells *in vitro*, PD led to a dose-dependent decrease in white blood cell count *in vivo* (Figure 8C). Subsequent studies revealed a significant upregulation of hemoglobin and erythropoietin expression in mice following treatment with PD (Figures 8D, E).

## 4 Discussion

AML, characterized by pronounced drug resistance and high mortality rates, poses a significant challenge in oncology (Chen et al., 2022; Stelmach and Trumpp, 2023). Identifying and developing innovative therapeutics is essential to combat the pressing issue of AML. This study investigated the therapeutic efficacy and underlying mechanisms of PD in AML. The findings in this study indicated that PD potently inhibited the proliferation of AML cells *in vitro*, induced apoptosis, and diminished inflammatory responses. The results demonstrated that PD effectively inhibited the proliferation of AML cells *in vitro*, induced apoptosis, and reduced inflammatory markers. Furthermore, *in vitro* studies revealed a substantial decrease in tumor growth and WBC count after treatment with PD. Moreover, histopathological assessments of vital organs, including the heart, liver, kidneys, spleen, and lungs, exhibited no abnormalities post-treatment, and body weight changes were comparable between the PD-treated group and the control group. In addition, PD increased Hb and Epo levels in mice after treatment, indicating PD did not had myelosuppression effects, which is a commonly seen side effects of AML therapy. These findings suggest a lack of discernible toxicity and adverse effects of PD on the mice. This research presents the initial evidence of PD’s anti-tumor potential specifically in the context of AML, highlighting its promise as a novel therapeutic candidate.

The pathological process of AML is complicated, involving the defects of the immune system and the disorder of cell metabolism (Cai and Levine, 2019; Ayyadurai et al., 2022). Autophagy, as a cellular self-cleaning mechanism, is crucial for maintaining cellular homeostasis (Debnath et al., 2023). It has been shown that the macrolide antibiotic brefeldin A (BFA) triggers endoplasmic reticulum stress-mediated expression of binding immunoglobulin (such as Bip) in colorectal cancer cells, leading to a decrease in Akt phosphorylation, which activates autophagy and ultimately apoptosis (Zhou et al., 2019). Similarly, estrogen receptor beta has been shown to suppress breast cancer cell migration and invasion through CLDN6-mediated autophagy (Song et al., 2019). Notably, inhibition of autophagy has been associated with increased apoptosis in various tumor types, including hepatocellular carcinoma and non-small cell lung carcinoma (Wu et al., 2021; Xue et al., 2022; Lin et al., 2021), thereby exerting anti-tumor effects. This suggests that autophagy may be heterogeneous in different tumors. In this study, PD was found to mitigate the progression of AML by reducing the ubiquitination of ATG5, thereby enhancing its protein stability and activating autophagy-related pathways that promote apoptosis in AML cells.

ATG5 is one of the key proteins in the autophagy pathway, serving as a biomarker for the initiation and progression of autophagy (Hamasaki et al., 2013). During the process of autophagy, ATG5 and ATG12 undergo covalent binding to form the ATG5-ATG12 complex (Okai et al., 2022). This complex subsequently interacts with ATG16 to yield the ATG5-ATG12-ATG16 complex (Fang et al., 2022). The assembly of this consortium complex plays a pivotal role in the initial stages of autophagic vesicle membrane formation, which supports the maturation of autophagic vesicles (Simon and Friis, 2014; Romanov et al., 2012). Evidence suggested that the suppression of ATG5-mediated autophagy may contribute to the development and progression of breast cancer (Liang et al., 2021). This study elucidated that PD suppresses the proliferation of AML cells and triggers apoptosis by activating ATG5-mediated autophagy. Specifically, ATG5 may regulate exogenous apoptotic pathways by competitively binding to Fas-associated proteins with death domains (FADD), thereby blocking the interaction between FADDs and death-inducing signaling complexes, which trigger exogenous apoptotic signaling pathways (Pyo et al., 2005). Yousefi et al. proposed that overexpression of ATG5 increases the sensitivity of cells to chemotherapeutic drugs, while knockdown of ATG5 reduces drug-induced apoptosis (Yousefi et al., 2006). However, the downregulation of ATG5 did not impact FADD-dependent cell death, and its inhibition of cysteine asparaginase expression did not influence autophagosome formation (Luo and Rubinsztein, 2007), which suggests that ATG5 may play a role in both autophagy and certain specific cell death pathways, yet these two biological processes can function independently of each other.

In conclusion, this research has established that PD ameliorated the pathogenesis of AML through the modulation of autophagy, specifically by inhibiting the ubiquitination of ATG5. Given the potent tumor-suppressive effects and low toxicity, PD emerges as a promising candidate for AML treatment. In future studies, the evaluation of the efficacy of PD in AML should be further strengthened in the clinic, with a view to providing a stronger basis for the treatment of AML in the clinic.

## Data Availability

The original contributions presented in the study are publicly available. This data can be found here: Dryad repository, accession DOI: 10.5061/dryad.d7wm37q9f, https://datadryad.org/stash/dataset/doi:10.5061/dryad.d7wm37q9f/.

## Abbreviations

AML, Acute myeloid leukemia; LSCs, leukemic stem cells; IL-1β, Interleukin-1β; TNF-α, Tumor necrosis factor-α; IL-6, Interleukin-6; ATG5, Autophagy-related protein 5; ATG7, Autophagy-related protein 7; LC3, Microtubule-associated protein 1A/1B-light chain 3; PD, Polydatin; Akt, Protein Kinase B; B-cell, B lymphocyte; BCL2, B cell lymphoma 2; EDU, 5-bromo-2-deoxyuracil; BAX, Bcl-2 Associated X protein; PARP, Poly ADP-ribose polymerase; CCK-8, Cell counting kit-8; cDNA, Complementary DNA; PAGE, Polyacrylamide gel electrophoresis; SDS, Sodium dodecyl sulfate; BCA, Protein Quantification Kit; PVDF, Polyvinylidene fluoride; WBC, White blood cell count; Hb, Hemoglobin; Epo, Erythropoietin; HE, Hematoxylin-eosin staining; IC50, 50%inhibitoryconcentration; BFA, Brefeldin A.
