# Supplementary material for: Targeting autophagy: polydatin’s role in inducing cell death in AML
Source: Front Pharmacol. 2024 Nov 19;15:1470217. doi: 10.3389/fphar.2024.1470217 (PMC11613146; doi:10.3389/fphar.2024.1470217)
Supplement: Supplementary file 1 [file Table1.docx]

Supplementary Material

# Supplementary Tables

Supplementary Table S1: ATG5 primer sequence

| *Homo sapiens* | Forward Primer | AAAGATGTGCTTCGAGATGTGT |
| --- | --- | --- |
|  | Reverse Primer | CACTTTGTCAGTTACCAACGTCA |
| *Mus musculus* | Forward Primer | TGTGCTTCGAGATGTGTGGTT |
|  | Reverse Primer | ACCAACGTCAAATAGCTGACTC |
